# Mixed Shock due to Sepsis and Anaphylactic Reaction With Kounis Syndrome Secondary to Dipyrone (Metamizole): Case Report

**DOI:** 10.1155/crcc/2519508

**Published:** 2026-05-14

**Authors:** Anthony Pellon

**Affiliations:** ^1^ Department of Medicine, Hospital II Huamanga EsSalud, Ayacucho, Peru; ^2^ Faculty of Health Sciences, Universidad Nacional San Cristóbal de Huamanga, Ayacucho, Peru

**Keywords:** anaphylaxis, dipyrone, Kounis syndrome, sepsis, shock

## Abstract

Kounis syndrome (KS) is a rare condition that combines acute coronary syndrome (ACS) with an allergic or anaphylactic reaction; in this case, it also occurred alongside sepsis, a coexistence not previously reported in scientific literature. The case of a 65‐year‐old man is presented with a history of allergy to penicillin and metamizole, admitted for persistent fever. Following intravenous metamizole administration, he developed hypotension, chest pain, bradycardia, dynamic electrocardiographic changes, and elevated troponin levels, in the context of pneumonia and hematologic dysfunction suggestive of sepsis. The final diagnosis was mixed shock (anaphylactic and septic) with Type I KS. Intramuscular epinephrine, antiallergic therapy, antibiotics, and ACS management were administered, with favorable evolution. This case underscores the importance of recognizing KS, an underdiagnosed entity, even in scenarios of critical comorbidity, as well as the need to maintain a broad differential diagnosis in the emergency department to prevent potentially serious diagnostic delays and errors.

## 1. Introduction

The KS is described as the occurrence of ACS triggered by the activation of mast cells and platelets (plt) during an allergic reaction or an anaphylactic episode. Pathophysiologically, it is classified into three types: Type I, which is coronary spasm due to an allergic reaction in arteries without preexisting disease; Type II, which occurs in individuals with prior atherosclerosis where the allergic reaction, in addition to spasm, ruptures or erodes the atherosclerotic plaque, leading to acute myocardial infarction (AMI); and Type III, which is associated with stent thrombosis or restenosis due to allergic inflammation, later confirmed in autopsies showing infiltration of eosinophils and mast cells [1].

Sepsis is a potentially life‐threatening condition in which organ dysfunction occurs because of infection, and septic shock is the undesirable progression of septic disease, characterized by marked tissue hypoperfusion, further worsening the patient′s severity [2].

Anaphylactic shock is a very severe, potentially fatal allergic reaction with rapid onset, causing massive vasodilation and increased capillary permeability mediated by the release of inflammatory mediators, leading to tissue hypoperfusion with vital organ compromise [3].

**Figure 1:**
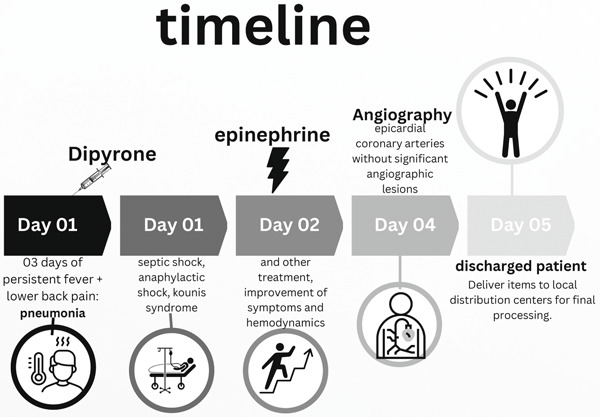
Clinical timeline.

Diagnostic error is a mistake in clinical interpretation that may delay or prevent appropriate treatment, jeopardizing the patient′s health and life [4]. The clinical course of the patient is summarized in Figure 1.

## 2. Case

A 65‐year‐old male, born in Ayacucho, Peru, and working as a lawyer, with a history of anaphylactic shock due to penicillin 20 years ago, appendectomy 17 years ago, hospitalization for spondylodiscitis 16 years ago, arterial hypertension treated with losartan 50 mg/day for the last 6 years, hospitalization for severe COVID‐19 pneumonia 5 years ago, kidney stones, and allergies to penicillin and dipyrone. Three days before emergency admission, he was evaluated on an outpatient basis, diagnosed with bronchitis, and prescribed lincomycin, prednisone, montelukast, and inhalation with formoterol/fluticasone.

He was presented to the emergency department with persistent fever and low back pain. After the intravenous administration of 1 g of metamizole, the patient immediately developed blurred vision, a sensation of faintness, retrosternal oppressive chest pain radiating to the left upper limb (8/10 on the visual analogue scale), and cold sweating, leading to admission to the trauma shock unit.

### 2.1. Clinical Findings

At the initial emergency assessment, the patient had a blood pressure (BP) of 121/98 mmHg, heart rate (HR) 86 bpm, peripheral oxygen saturation measured by pulse oximetry (SpO_2_) 94%, temperature 36.4°C, and bilateral positive fist percussion.

Following dipyrone administration, the patient′s BP dropped to 70/40 mmHg, HR to 56 bpm, respiratory rate to 30 breaths per minute, temperature was 37.0°C, SpO_2_ 95%, Glasgow Coma Scale 15/15, with diaphoresis and crackles at the bases of both lungs.

### 2.2. Diagnostic Assessments

After the first evaluation, the patient presented the following results: white blood cell count (WBC) 4470 cells/mm^3^, segmented neutrophils (segs) 77.7%, lymphocytes (lymph) 17.7%, plt 122,000 cells/mm^3^, mean platelet volume (MPV) 10.0 fL, considered elevated (upper limit is 9.5 fL), urine sediment WBC 10–12 per field, red blood cells 20–25 per field, C‐reactive protein (CRP) 4.62 mg/dL, COVID‐19 antigen nonreactive, urine culture negative.

**Figure 2:**
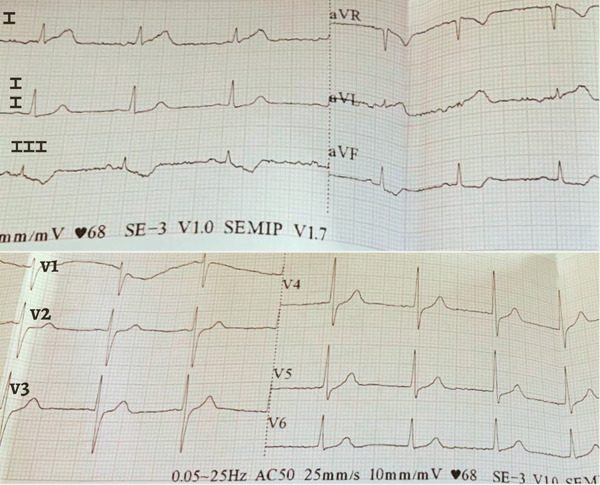
ECG at symptom onset showing ST‐segment depression in Leads III and aVF.

**Figure 3:**
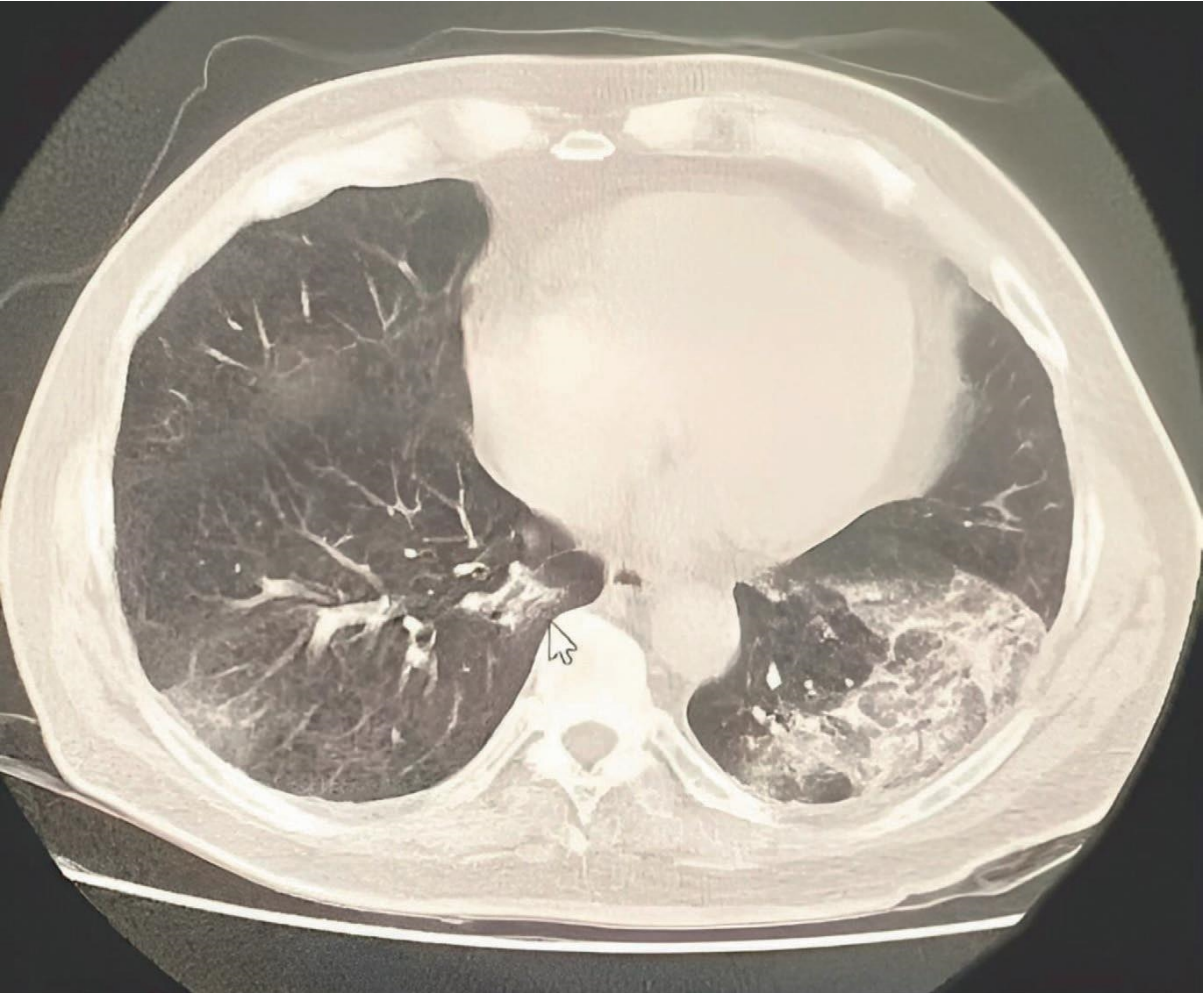
Noncontrast CT scan from the day of metamizole exposure.

Following shock and precordial pain, the patient presented: WBC 4,530 cells/mm^3^, segs 82%, lymph 14%, plt 120,000 cells/mm^3^, MPV 10 fL, albumin 3.31 g/dL, creatinine 1.36 mg/dL, urea 63 mg/dL, creatine kinase MB fraction (CK‐MB) 19.9 U/L, high‐sensitivity troponin T (hs‐TnT) 0.003 ng/mL. Electrocardiogram (ECG) (Figure 2) showed 1 mm ST depression in Leads III and aVF. Noncontrast CT scan (Figure 3) revealed some alveolo‐interstitial opacities in both lower lung lobes, with a tendency toward consolidation on the left side, suggestive of an inflammatory process.

**Figure 4:**
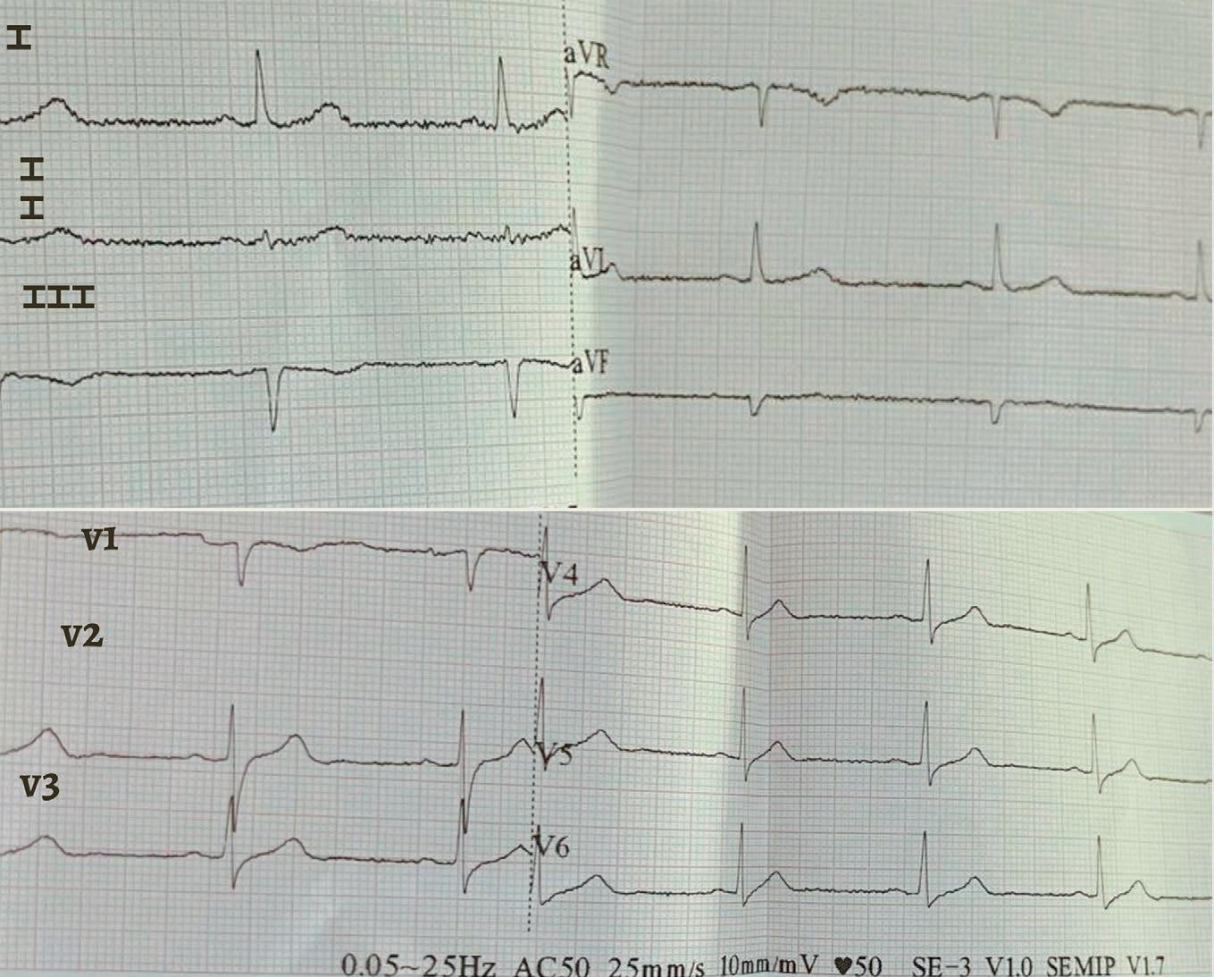
Subsequent ECG showing QS complexes in Leads III and aVF.

The following day, results were: hs‐TnT > 50 ng/mL, WBC 4,320 cells/mm^3^, segs 65.8%, lymph 32.3%, plt 123,000 cells/mm^3^, urine sediment WBC 6–8 per field, red blood cells 0–1 per field, creatinine 0.98 mg/dL, urea 52 mg/dL, procalcitonin 0.09 ng/mL; ECG (Figure 4) showed QS waves in leads III and aVF.

On the next morning, upon arrival at the referral hospital for coronary angiography, the following were found: hs‐TnT 2.025 ng/mL, CK‐MB 23.5 U/L, WBC 3,740 cells/mm^3^, segs 67%, lymph 27.6%, plt 129,000 cells/mm^3^, creatinine 1.0 mg/dL, urea 46 mg/dL, urine culture negative. That night: hs‐TnT 1.633 ng/mL, CK‐MB 6.4 U/L.

**Figure 5:**
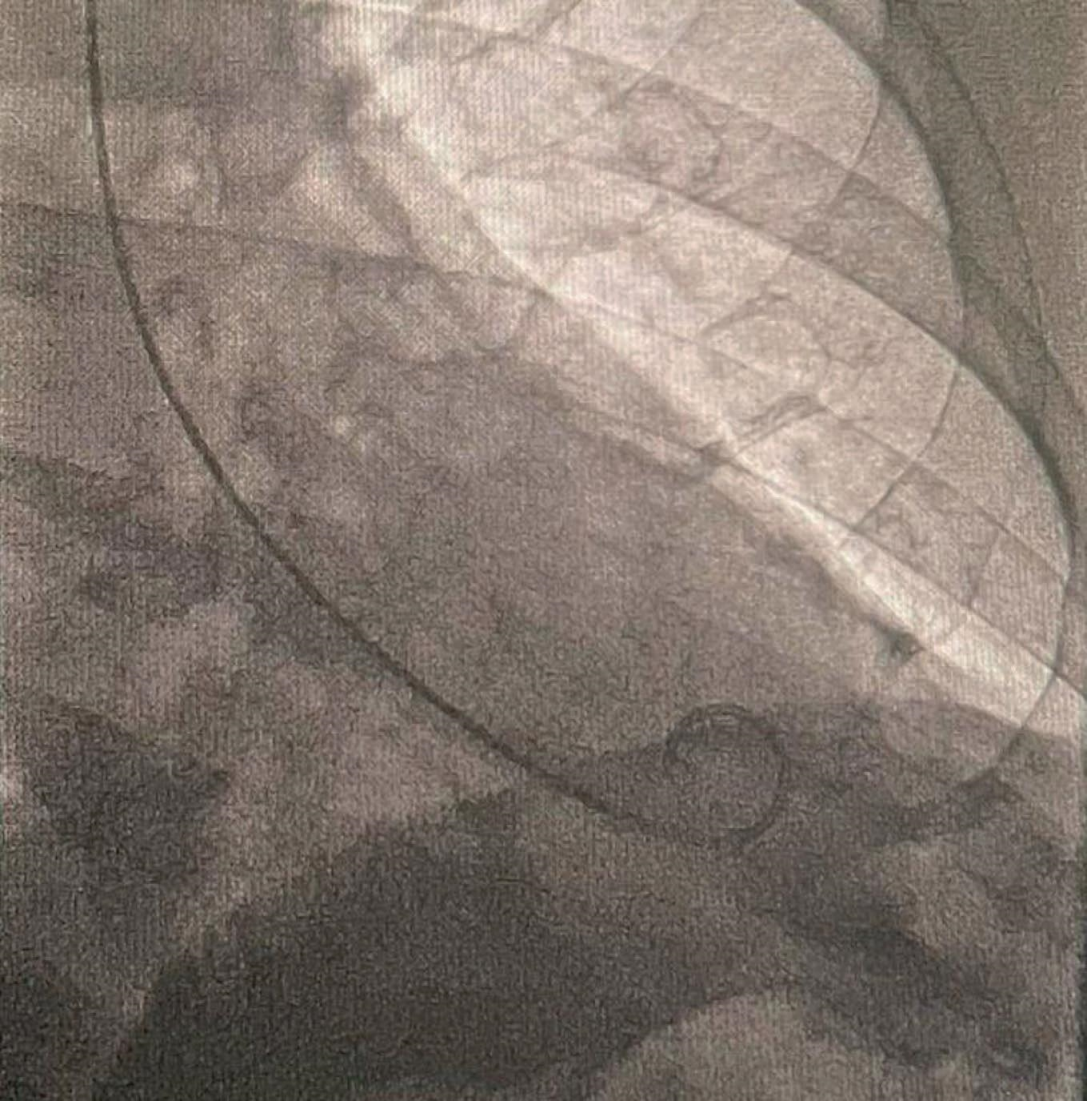
Coronary angiography on hospital day four showing no evidence of any lesion.

The following night, coronary angiography (Figure 5) was performed, revealing a dominant circumflex artery and epicardial coronary arteries without significant angiographic lesions. Additionally, hs‐TnT 1.366 ng/mL, CK‐MB 3.9 U/L, WBC 3,760 cells/mm^3^, segs 56%, lymph 32.6%, plt 172,000 cells/mm^3^, CRP 2.1 mg/dL, and procalcitonin 0.086 ng/mL.

Initially, upon admission to the trauma shock unit, where the patient was managed overnight by a primary care physician, the following diagnoses were made: non‐MI angina pectoris, nonpharmacological hypotension, dipyrone‐induced diaphoresis, generalized anxiety disorder.

Subsequently, still unstable, the patient was transferred to the observation unit where the next day, he was attended by a physician certified in initial critical care management, who diagnosed: anaphylactic shock, acute respiratory failure, inferior wall myocardial infarction, KS, and pneumonia.

He was hospitalized for 5 days and discharged asymptomatic and without sequelae.

### 2.3. Therapeutic Interventions

At the onset of shock and AMI, before they were diagnosed as such, 500 mL of normal saline (NS) was administered by direct intravenous (IV) injection and 500 mL of 3.5% gelatin‐type colloid solution. As maintenance therapy: 1000 mL NS + 20*%* KCl (one ampoule) at 75 cc/h, diazepam 5 mg by direct IV injection, chlorphenamine 10 mg IV every 8 h, metoclopramide 10 mg IV every 8 h, amikacin 1 g IV every 24 h, O_2_ at 2 L/min.

Subsequently, once KS and mixed shock were diagnosed, the following treatment was given 1000 mL NS + 20*%* KCl (1 ampoule) at 80 cc/h, epinephrine 1/10000 5 mL intramuscular immediately, and then as needed for bronchospasm, shock, and/or other signs of anaphylaxis; amikacin 1 g IV every 24 h, ipratropium bromide 20 *μ*g with three inhalations every 6 h, omeprazole 40 mg IV every 24 h, enoxaparin 60 mg subcutaneous every 12 h, atorvastatin 80 mg orally (PO) immediately, cetirizine 10 mg PO every 12 h, clopidogrel 300 mg PO immediately, acetylsalicylic acid 300 mg PO immediately, hydrocortisone 100 mg IV every 8 h, oxygen therapy to maintain SpO_2_ at 94%.

The patient was discharged with acetylsalicylic acid 100 mg PO every 24 h, atorvastatin 40 mg PO every 24 h, and azithromycin 500 mg PO every 24 h.

## 3. Discussion

This case describes a rare and clinically complex presentation of KS Type I occurring in the context of a severe anaphylactic reaction induced by metamizole, superimposed on a probable septic state secondary to pneumonia. To the best of our knowledge, this is the first reported case describing the simultaneous coexistence of KS, sepsis, and anaphylactic shock, representing a unique and diagnostically challenging scenario.

KS is an underdiagnosed condition, with a variable prevalence ranging from 0.002% among patients undergoing catheterization in hemodynamics laboratories to 1.1% of patients hospitalized for allergic reactions. Likewise, most reported cases originate from countries such as Turkey, Greece, Italy, and Spain [5].

In KS, mast cell activation and degranulation, with the release of mediators such as histamine, tryptase, leukotrienes, and prostaglandins, induce coronary vasospasm, platelet activation, and endothelial dysfunction. At the molecular level, histamine promotes vasoconstriction and platelet aggregation, whereas tryptase and other mast cell proteases activate the coagulation cascade and matrix metalloproteinases, contributing to plaque instability and a prothrombotic state [1, 5, 6]. In parallel, during a septic state, there is a release of proinflammatory cytokines such as TNF‐*α* and IL‐1, which promote a procoagulant endothelial phenotype. Additionally, microvascular dysfunction develops, characterized by increased expression of adhesion molecules, leukocyte infiltration, glycocalyx damage, release of reactive oxygen species, and interaction between the coagulation and complement systems, leading to alterations in vascular permeability, vascular tone, and tissue perfusion [7]. IL‐6 contributes to the amplification of the systemic inflammatory response and progression of organ dysfunction; furthermore, sepsis also induces myocardial dysfunction mediated by inflammatory cytokines, oxidative stress, and cellular alterations, resulting in reduced contractility and perpetuation of shock [8]. Altogether, a potential synergistic interaction between mast cell mediators and proinflammatory cytokines creates a milieu of vasospasm, immunothrombosis, microvascular dysfunction, and myocardial depression, representing a key integrative mechanism underlying the severity of KS in the context of sepsis.

N‐(2,3‐dimethyl‐5‐oxo‐1‐phenyl‐3‐pyrazolin‐4‐yl), or metamizole, acts as a prodrug and exhibits high water solubility, an acidity level close to neutral, and low plasma protein binding, which favors its rapid systemic distribution. When administered intravenously, the parent compound is detectable in the bloodstream for approximately 15 min. Although its complete mechanism of action remains unclear, based on the characteristics, it can be considered a rapidly acting prodrug [9].

Metamizole‐induced hypersensitivity reactions comprise both nonimmunological and immunological mechanisms. Nonallergic reactions are typically related to cyclooxygenase inhibition and subsequent leukotriene overproduction, leading to cross‐intolerance with multiple non‐steroidal anti‐inflammatory drugs (NSAIDs). In contrast, selective reactions to metamizole are considered immunologically mediated, with immediate reactions predominantly driven by specific IgE antibodies and delayed reactions involving T‐cell–mediated responses. Emerging evidence suggests that metamizole metabolites, particularly 4‐methylaminoantipyrine, play a crucial role in the immune response. Indeed, the inclusion of these metabolites in basophil activation tests significantly increases diagnostic sensitivity, supporting the hypothesis that metabolites rather than the parent compound act as antigenic determinants in selective anaphylaxis [10, 11].

Regarding frequency and severity, NSAIDs represent the most common class of drugs involved in hypersensitivity reactions overall. Within this group, pyrazolones such as metamizole are among the most frequently implicated in selective immediate reactions. In a large retrospective cohort, anaphylaxis was identified in approximately one‐third of patients with confirmed metamizole hypersensitivity, with most cases presenting moderate to severe clinical manifestations and rapid onset within the first hour after drug administration [10]. Although direct comparative data between individual NSAIDs is limited, these findings highlight that metamizole‐induced anaphylaxis is not uncommon and may be clinically severe.

The coexistence of hypersensitivity to both penicillin and metamizole in the same patient may suggest an underlying predisposition to drug hypersensitivity rather than cross‐reactivity. These drugs belong to distinct pharmacological classes but are among the most frequently implicated in immune‐mediated drug reactions. Clinical studies have shown that a substantial proportion of patients evaluated for drug allergy report reactions to multiple active substances, supporting the concept of an individual susceptibility to drug sensitization [12]. Therefore, the presence of multiple drug allergies in this patient may reflect a broader hypersensitivity phenotype, warranting careful evaluation and long‐term pharmacovigilance.

In this case, the electrocardiographic evolution from ST‐segment depression to QS waves can be explained not only by the pathophysiological mechanisms of Kounis syndrome but also by the prolonged duration of untreated ischemia. The initial release of mast cell mediators, including histamine and leukotrienes, likely induced coronary vasospasm and endothelial dysfunction, resulting in subendocardial ischemia and ST‐segment depression. However, the absence of early targeted treatment for both anaphylaxis and myocardial ischemia, with a delay exceeding 12 h, likely allowed the persistence of coronary vasospasm and microvascular dysfunction. This sustained reduction in coronary perfusion likely led to progression from reversible ischemia to myocardial necrosis, explaining the development of QS waves despite the absence of obstructive coronary lesions on angiography [1, 6]. This temporal progression reinforces the critical role of early recognition and prompt treatment in preventing irreversible myocardial injury.

The septic state of the patient is inferred from evidence of hematological organ dysfunction, demonstrated by thrombocytopenia and increased MPV [13], rather than by the traditional classification of two or more points in the SOFA score [2], and this was recorded before dipyrone was administered. After the administration of dipyrone, the patient developed shock due to a mixed component: the main one, by virtue of its rapid onset, was anaphylactic [3], based on a predisposed septic state. Both components share the same primary pathophysiology causing shock, although not the only one: a significant drop in peripheral vascular resistance [3, 7], which possibly acted synergistically to produce severe hemodynamic instability characterized by shock and inappropriate bradycardia [7, 14]. Other reports have shown the coexistence of septic and anaphylactic shock; however, this unprecedented report also demonstrates the simultaneous presence of KS [15].

Type I KS in this patient was evidenced by the clinical picture consistent with myocardial infarction secondary to a severe allergic reaction, and the absence of atherosclerotic lesions in the coronary angiography [1]. The decision to administer intramuscular epinephrine was based on a risk–benefit assessment in the setting of life‐threatening anaphylaxis with persistent hemodynamic instability. Current anaphylaxis practice parameters identify epinephrine as the first‐line treatment and emphasize that its administration should not be delayed in severe reactions. Intramuscular administration is preferred over intravenous administration because it is associated with a lower risk of dosing errors and serious adverse events, which are rare when administered appropriately [14].

Despite the theoretical concern that epinephrine may exacerbate coronary vasospasm in Kounis syndrome, the immediate threat posed by the anaphylactic component of the shock outweighed this potential risk. In this case, the patient remained hemodynamically unstable for more than 12 h, presenting persistent chest pain, hypotension, and bradycardia prior to epinephrine administration. Following treatment, a rapid improvement in BP was observed within approximately 5 min and went from 70/40 mmHg (lowest values) to 101/64 mmHg, consistent with reversal of distributive shock. Complete clinical stabilization, including normalization of HR, respiratory rate, and resolution of most symptoms, was achieved within approximately 4 h.

Although the patient had a favorable outcome without any sequelae, it is noteworthy that at the outset the coexistence of these diseases was not identified for definitive treatment, in an emergency context where there are also time and resource constraints that may favor diagnostic errors, which could have posed a life‐threatening risk to the patient [4].

This case illustrates how diagnostic delay in complex shock states may lead to significant myocardial injury, even in the absence of obstructive coronary artery disease. It also highlights the importance of timely management of anaphylaxis, in which the administration of intramuscular epinephrine can be life saving and should not be delayed.

## Author Contributions

**Anthony Pellon:** conceptualization, investigation, drafting – original draft, drafting – review and editing, visualization, supervision, project administration.

## Funding

No funding was received for this manuscript.

## Disclosure

A preprint has previously been published [16].

## Consent

Informed consent was obtained from the patient for the anonymous use of clinical information for academic and publication purposes.

## Conflicts of Interest

The author declares no conflicts of interest.

## Patient Perspective

The patient stated that at first, he felt very worried due to the chest pain and sensation of faintness, but became calmer as his symptoms improved and, especially upon learning after the cardiac catheterization, that his heart had no damage. Two months later, he considers the episode a serious but anecdotal event from which he survived and for which he feels grateful.

## Data Availability

The data that support the findings of this study are available on request from the corresponding author. The data are not publicly available due to privacy or ethical restrictions.
